# Real‐Time Eco–AI, Electrophoresis‐Correlative Data‐Dependent Acquisition with AI‐Based Data Processing Broadens Access to Single‐Cell Mass Spectrometry Proteomics

**DOI:** 10.1002/anie.202510692

**Published:** 2025-08-23

**Authors:** Bowen Shen, Fei Zhou, Peter Nemes

**Affiliations:** ^1^ Department of Chemistry & Biochemistry, University of Maryland College Park MD 20742 USA

**Keywords:** Capillary electrophoresis, Intelligent data acquisition, Mass spectrometry, Proteomics, Single cell

## Abstract

Single‐cell mass spectrometry (MS) offers unprecedented sensitivity for profiling cellular proteomes, yet widespread adoption is hindered by the cost of advanced instrumentation. Here, we broaden access to single‐cell proteomics by combining capillary electrophoresis (CE), data‐dependent acquisition (DDA) with electrophoresis‐correlative (Eco) ion sorting, and artificial intelligence (AI)‐assisted spectral deconvolution via CHIMERYS (Eco–AI). This “Real‐Time Eco–AI” workflow was implemented on a custom‐built CE platform coupled to a legacy hybrid quadrupole‐orbitrap mass spectrometer (Q Exactive Plus). Despite slower scan speed, lower resolution, and inferior ion transmission efficiency, real‐time Eco‐DDA sampling and CHIMERYS processing enabled identification of up to ∼15 peptides per spectrum—performance on par with modern Orbitrap Fusion Lumos tribrid systems. From 1 ng of HeLa digest, 2142 proteins were identified, surpassing the 969 proteins detected on a contemporary nanoLC Orbitrap Fusion Lumos. Even from ∼250 pg (a single‐cell equivalent), 1799 proteins were identified in <15 min of effective separation, raising a theoretical throughput of 48 samples per day. As proof of principle, Real‐Time Eco–AI profiled 1524 proteins from single precursor cells (50–75 µm diameter) in *Xenopus laevis* blastulae, revealing proteome asymmetry during neural versus epidermal fate specification. These results establish Real‐Time Eco–AI as a budget‐conscious yet powerful strategy for single‐cell proteomics using CE–MS.

## Graphical abstract

An affordable single‐cell proteomics workflow combines capillary electrophoresis, real‐time ion sorting, and AI‐aided spectral deconvolution to profile early *Xenopus* embryonic cells. Despite using a legacy Orbitrap, this strategy matches or exceeds the sensitivity of modern instruments, uncovering proteome dynamics at sub‐nanogram scale.

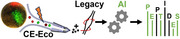

## Introduction

Single‐cell mass spectrometry (MS) is at the forefront of modern analytical science. Active technical development seeks to expand the detectable proteome by isolating single cells, processing their protein content, and analyzing it with ever‐greater efficiency (reviewed in Refs. [1, 2]). Short analytical times are particularly desirable to enhance statistical power by enabling the analysis of entire cell populations, one cell at a time. Since no amplification strategy exists for whole proteomes, access to mass spectrometers with exceptional sensitivity, resolution, and speed is critical for achieving deep proteomic coverage.

Single‐cell MS integrates numerous technological advances. Early works using capillary electrophoresis (CE)–MS on hemoglobin in individual erythrocytes^[^ ^3^, ^4^ ^]^ and high‐sensitivity analyses^[^ ^5^, ^6^, ^7^, ^8^ ^]^ laid the foundation for the field. CE–MS enabled one of the first demonstrations of single‐cell proteomics in large‐to‐mid‐sized (∼250–75 µm diameter) embryonic stem cells (blastomeres) dissected manually.^[^ ^9^ ^]^ Despite the overwhelming ∼90% yolk protein content in these cells during early development,^[^ ^10^, ^11^ ^]^ improvements such as simplified sample preparation workflows^[^ ^12^ ^]^ and nanoLC–MS integration^[^ ^13^, ^14^ ^]^ increased the measurable proteome fraction to whole cells. The adoption of automated cell sorters extended single‐cell MS to high‐throughput analysis of smaller (∼10–75 µm) cells. Nanoliter‐scale processing platforms such as nanoPOTS^[^ ^15^ ^]^ and nPOP^[^ ^16^ ^]^ further miniaturized bottom‐up workflows to sub‐microliter volumes. Chemical barcoding significantly increased throughput,^[^ ^12^ ^]^ ultimately enabling today's high‐throughput single‐cell MS via nanoLC.^[^ ^17^ ^]^ Advances in separation, for example through fast nanoLC,^[^ ^18^ ^]^ CE,^[^ ^19^ ^]^ and ion mobility,^[^ ^20^, ^21^ ^]^ reduced effective analysis times to below 15 min, making single‐cell MS increasingly viable in clinical contexts.^[^ ^22^ ^]^ These reduced timeframes, however, demand exceptional speed, sensitivity, and resolution for detection and quantification, further calling for top‐of‐the‐line instruments capable of meeting these technical needs.

Progress in data acquisition and analysis helped partially remedy these demands in CE–MS. In bottom‐up workflows, proteins are quantified via the sequencing of proteotypic peptides in a controlled and reproducible manner. Our laboratory^[^ ^23^, ^24^, ^25^ ^]^ and others^[^ ^26^, ^27^, ^28^ ^]^ initially used data‐dependent acquisition (DDA) to identify up to ∼1700 proteins from individual *Xenopus laevis* (South African clawed frog) blastomeres in 60‐min analyses.^[^ ^25^, ^29^ ^]^ Prioritized DDA, which ranks precursor ions by abundance, improved MS/MS utilization and identified ∼35% more proteins in limited mouse neuronal proteomes than the standard DDA.^[^ ^30^ ^]^ Data‐independent acquisition (DIA), by co‐fragmenting multiple peptide features within broad *m*/*z* windows, expanded empirical coverage to ∼1600 proteins from ∼130‐µm *X. laevis* blastomeres (∼6 nL cytoplasm)^[^ ^13^ ^]^ and to ∼2000–3000 proteins in mammalian cells^[^ ^21^, ^31^, ^32^ ^]^ using next‐generation nanoLC–MS. DIA also enhanced CE–MS performance, yielding ∼1100 proteins from single HeLa cell‐equivalent samples and ∼1200 proteins from *X. laevis* blastomeres within ∼15 mins.^[^ ^19^ ^]^ Incorporating ion mobility added another ∼50% to protein counts.^[^ ^33^ ^]^ Additional developments included prioritized SCoPE, which ranks peptides by spectral purity and biological relevance to optimize MS^2^ sampling^[^ ^34^ ^]^ and Slice‐PASEF, which targets precursors based on ion mobility to quantify 1417 proteins in single HeLa‐equivalent samples.^[^ ^35^ ^]^ More recently, wide‐window acquisition (WWA) combined with the AI‐driven CHIMERYS algorithm on next‐generation nanoLC–MS platforms has yielded average proteome depths of ∼2000 proteins per single cell.^[^ ^36^, ^37^, ^38^ ^]^ Thus, single‐cell MS is now capable of deep proteome coverage, provided that a suitably advanced mass spectrometer is available.

This project seeks to broaden access to single‐cell MS by developing a cost‐effective alternative that matches the proteomic depth of leading platforms. We identified CE as an ideal separation method for this purpose; it is efficient, reproducible, and significantly more economical than modern nanoLC systems. Unlike complex solvent delivery systems requiring frequent maintenance, CE operates using robust, low‐cost power supplies, offering substantial long‐term savings. Building on our published protocols,^[^ ^12^, ^29^, ^39^ ^]^ we constructed and validated an “affordable” single‐cell CE–ESI–MS platform using readily available components. To further improve sensitivity and proteome depth, we recently introduced electrophoresis‐correlative (Eco) peptide sorting, which boosts DIA coverage by 38%, even while using less than half of the available *m*/*z*–migration time (MT) space.^[^ ^40^ ^]^ To reap additional sensitivity from a decade‐old but broadly accessible quadrupole Orbitrap system (Q Exactive Plus, QE+, Thermo), we proposed to address the current inefficiency in MS^2^ sampling in CE–MS by leveraging *m*/*z* evolution via Eco‐sorting in real time. As with any new technology, comprehensive validation was required to evaluate protein detection sensitivity and quantitation reproducibility using a benchmark proteome standard. To assess its biological utility, we employed Real‐Time Eco–AI to investigate proteome remodeling during lineage specification in *X. laevis* embryos, comparing neural and epidermal fates.

## Results and Discussion

**Figure 1:**
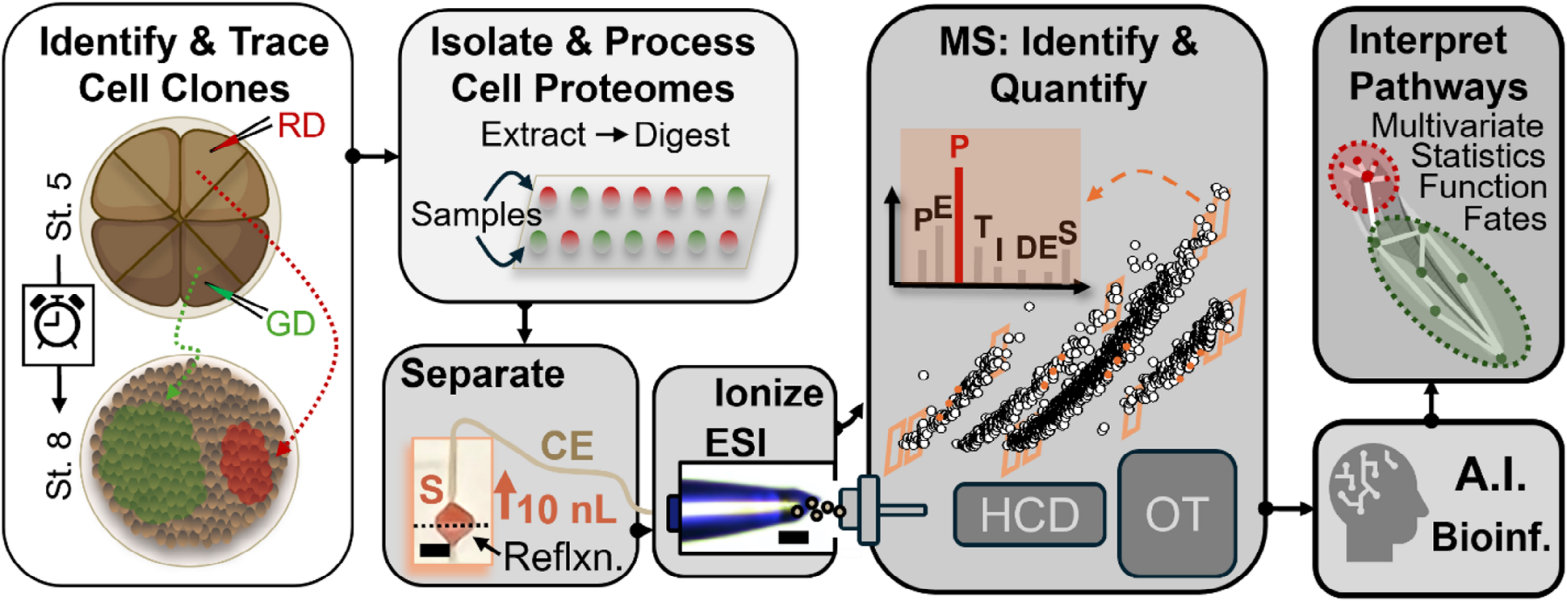
Strategy for affordable single‐cell proteomics using capillary electrophoresis (CE), electrospray ionization (ESI), and mass spectrometry (MS). Neural (D11) and epidermal (V11) precursor blastomeres of 16‐cell *Xenopus laevis* embryos (St. 5) were microinjected with red/green fluorescent dyes, tracked to the blastula stage (St. 8), and isolated for proteome analysis. CE was coupled to a legacy Orbitrap (Q Exactive Plus, Thermo) with electrophoresis‐correlative (Eco) ion sorting and real‐time data‐dependent acquisition (DDA). Artificial intelligence (CHIMERYS) resolved the resulting highly chimeric tandem mass spectra, achieving sensitivity comparable to modern tribrid instruments. D11 and V11 descendants were profiled to reveal proteome reorganization during early differentiation. (Created with BioRender.com).

Our objective was to deepen single‐cell proteome coverage using a cost‐efficient approach. The experimental strategy is outlined in Figure 1. As an alternative to nanoLC (reviewed in Refs. [1, 41, 42]), we strategically selected capillary zone electrophoresis (CE) for peptide separation. Our custom‐built CE–ESI platform, developed and validated as previously described,^[^ ^12^, ^29^ ^]^ offered ultrahigh sensitivity and reproducibility while substantially reducing acquisition, operational, and maintenance costs compared to nanoLC. We reused a legacy orbitrap mass spectrometer (Q Exactive Plus), which operates with reduced ion collection/transfer efficiency and slower spectral resolution (70 000 FWHM, 3 Hz tested here) compared to the modern alternatives (Fusion Lumos tested here at 120 000 FWHM) while representing a fraction of the cost. We sought to leverage Eco‐sorting^[^ ^40^, ^43^ ^]^ to simplify MS control by returning to the well‐established data‐dependent acquisition (DDA) mode available on most mass spectrometers. The resulting tandem MS data, highly chimeric due to isobaric co‐isolation, were analyzed using CHIMERYS (Thermo), an artificial intelligence (AI)‐powered software tool. The combined approach, termed **Eco–AI**, was validated using HeLa digests and benchmarked against a nanoLC–Orbitrap Fusion Lumos and Exploris systems. The technical details are available in the Methods in the Supporting Information document. We further applied Eco–AI to study proteome remodeling in dorsal‐animal midline (D11) and ventral‐animal midline (V11) blastomeres during neural and epidermal fate specification in *Xenopus laevis* embryos.

### Strategic Design Considerations for Eco–AI

**Figure 2:**
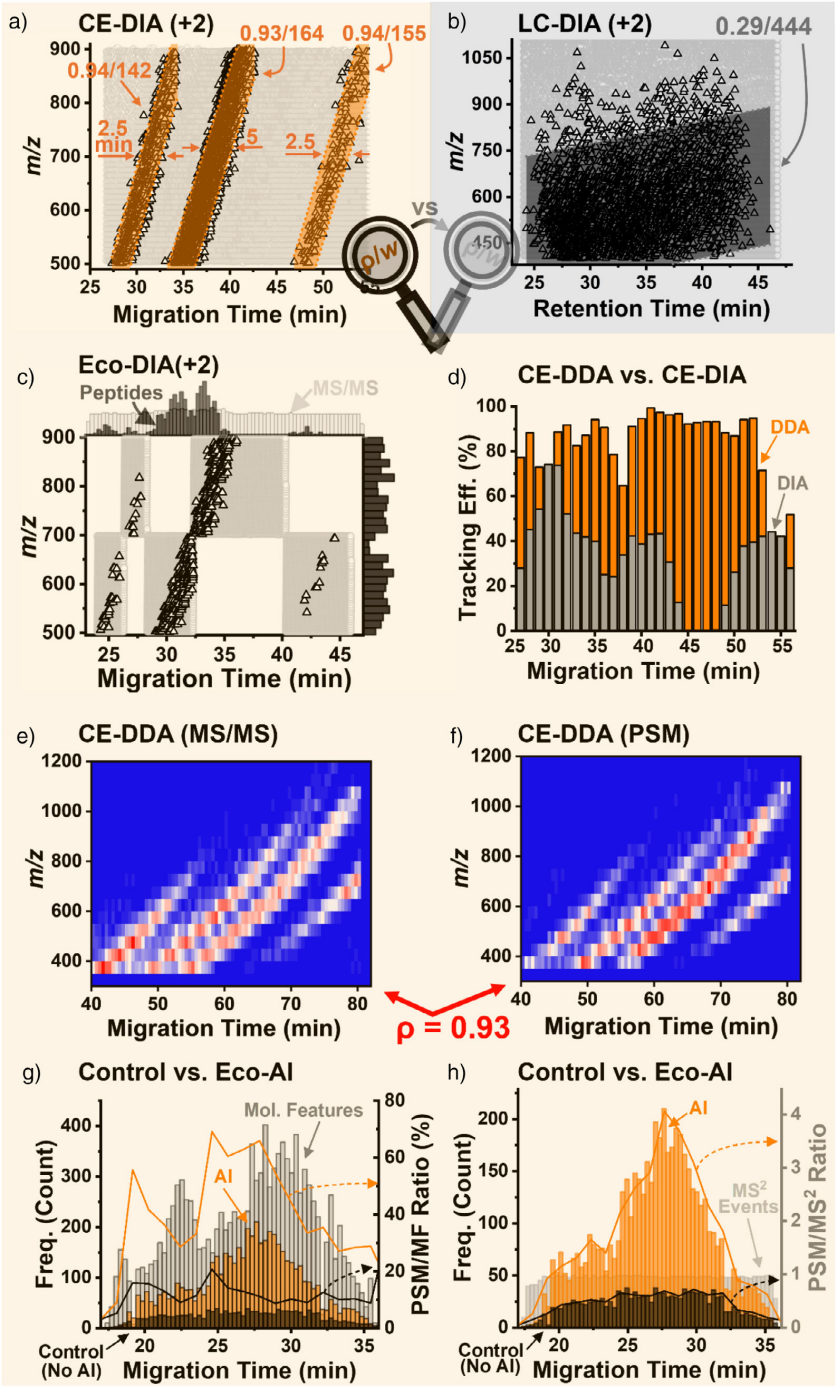
Advancing deeper proteomics through Real‐Time Electrophoresis‐Correlative (Eco) data acquisition with AI‐aided data processing on a legacy Orbitrap mass spectrometer. A 10 ng HeLa digest was analyzed by CE– and nanoLC–MS. a) Eco sorting grouped the peptide ions into temporally correlated data clusters based on mass‐to‐charge (m/z) and separation time (2+ charge shown, Pearson *ρ* ≥ 0.93), whereas b) nanoLC spread them broadly over the dimensions (*ρ* = 0.29). c) Eco‐data independent acquisition (DIA), using 5 segmented *m*/*z*–migration time windows, identified ∼38% more proteins than the control while using <50% of the analytical bandwidth. d) Data‐dependent acquisition (DDA) with standard isolation width (1.6 Th) tracked the *m*/*z*–migration correlation with up to 99% success. e), f) MS/MS events aligned with the density of the identified peptide spectral matches (PSMs), reflecting high real‐time sequencing efficiency (*ρ* = 0.93). g), h) AI‐assisted data processing software (CHIMERYS, Thermo) improved deconvolution of highly chimeric spectra, identifying over 3× more molecular features (MFs). Together, these results demonstrate that Real‐Time Eco–AI enables deep proteome coverage even on earlier‐generation Orbitrap instruments.

Our first goal was to identify the conditions enabling robust single‐cell proteomics using CE–MS on a legacy QE+ versus a modern nanoLC–Lumos and Exploris platforms. Figure 2 and the Supporting Information document summarize the underlying mechanistic differences. In preliminary DIA experiments, CE–ESI with the QE+ identified 1578 proteins from ∼10 ng of HeLa digest, compared to 1357 proteins via nanoLC‐ESI on the Lumos. Consistent with our previous findings,^[^ ^33^ ^]^ peptides in CE were Eco‐sorted into distinct, charge‐dependent *m*/*z*–migration time (MT) trends (Figure 2a), whereas such ordering was negligible in reversed‐phase nanoLC (Figure 2b). All charge states (above 2+) were measured experimentally (considered for fragmentation) to maximize proteome depth (see Figure S1), and data analysis included all the measured charge states. As the +2 charge state accounted for >80% of identifications, we limit the discussion to this charge state for simplicity.

The impact of these separation differences was gauged on the emerging peptide ion composition (Figure 2). In CE (Figure 2a), Eco‐sorting organized >95% of the precursor ions into 3 highly correlated *m*/*z*–MT clusters (Pearson *ρ* ≥ 0.93 each), with narrow widths (*w*) of 142 Th (∼27–34 min), 164 Th (∼34–43 min), and 155 Th (∼46–55 min). In contrast, nanoLC (Figure 2b) dispersed the peptide *m*/*z* values across a broad 444 Th window (*ρ* ∼ 0.29). These CE patterns created “hotspots” of high peptide spectral match (PSM) density, separated by low‐yield regions. Such structuring was unimpressive in nanoLC, where stochastic *m*/*z*–retention time (RT) signal evolution dominates. These data guided our working notion that classical data acquisition modalities optimized to nanoLC do not necessarily address high transient fluxes of isobaric signals (nominal m/z values) emerging during CE.

We sought to explore this CE‐specific signal behavior to tune up the MS sequencing efficiency. As shown in Figure 2c, we applied 5 fixed Eco–DIA windows^[^ ^40^ ^]^ (grey rectangle regions) to cover the *m*/*z* regions that contained peptide features (black triangles). This increased MS^2^ utilization by ∼50%, identifying 846 proteins from just 1 ng of digest. However, still <50% of the sampled *m*/*z*–MT space contained peptide signals, indicating inefficient bandwidth use. To improve tracking efficiency, we estimated peptide migration speed (∼1 Th s^−1^; Figure 2a) and used co‐migration characteristics to tailor the precursor isolation window (*w* = 2–4 Th windows^[^ ^44^ ^]^). Since most peptide feature abundances exceed background noise, even from single‐cell samples, we rationalized that DDA was ideally suited to track these trends in real time, forming the basis of **Eco–DDA**, or **Real‐Time Eco–MS**.

Figure 2d evaluates the resulting tracking efficiency. 100% tracking efficiency is the ideal condition where all MS/MS acquisitions occurred on the *m*/*z* regions that contain peptide features, whereas 0% tracking efficiency refers to a complete miss. While DIA (10 Th isolation) captured signals ∼45% of the time, narrow DDA windows (*w* = 1.6 Th) achieved up to 99% efficiency. The tandem MS scans dynamically sampled the *m*/*z*–MT space (Figure 2e), yielding PSMs (Figure 2f) with high correlation (*ρ* = 0.93). These results confirmed a highly efficient sampling of the Eco‐sorted peptides for sequencing using the DDA modality in real time.

The quality of MS^2^ sequencing was also examined. Only 10%–20% of peptide‐like molecular features (MFs) were confidently identified by SEQUEST, typically yielding either a single or no peptide identification per MS^2^ scan. Given CE's narrow *m*/*z* trends, isobaric co‐isolation led to chimeric spectra. Recent advances in artificial intelligence (AI)‐based data processing raised a potential to extract multiple peptide signatures from the chimeric data. The CHIMERYS algorithm^[^ ^45^ ^]^ successfully resolved these spectra, extracting up to 15 PSMs per scan with a Percolator q value below 1% false discovery rate (Figure 2g), quadrupling the peptide identification efficiency. Representative tandem MS spectra are annotated in Figure S2. The theoretical effective electrophoretic mobility calculated for these peptide identifications, tabulated in Table S1, was highly correlated with the apparent, the empirically determined values (*ρ* = 0.98, Figure S3), corroborating the accuracy of the identifications. On average, this AI‐assisted data processing tripled peptide identifications per spectrum compared to conventional analysis (Figure 2h). The AI‐based data analysis tool proved essential for decoding the complex CE–MS data.

### Performance Validation for Single‐Cell Proteomics

**Figure 3:**
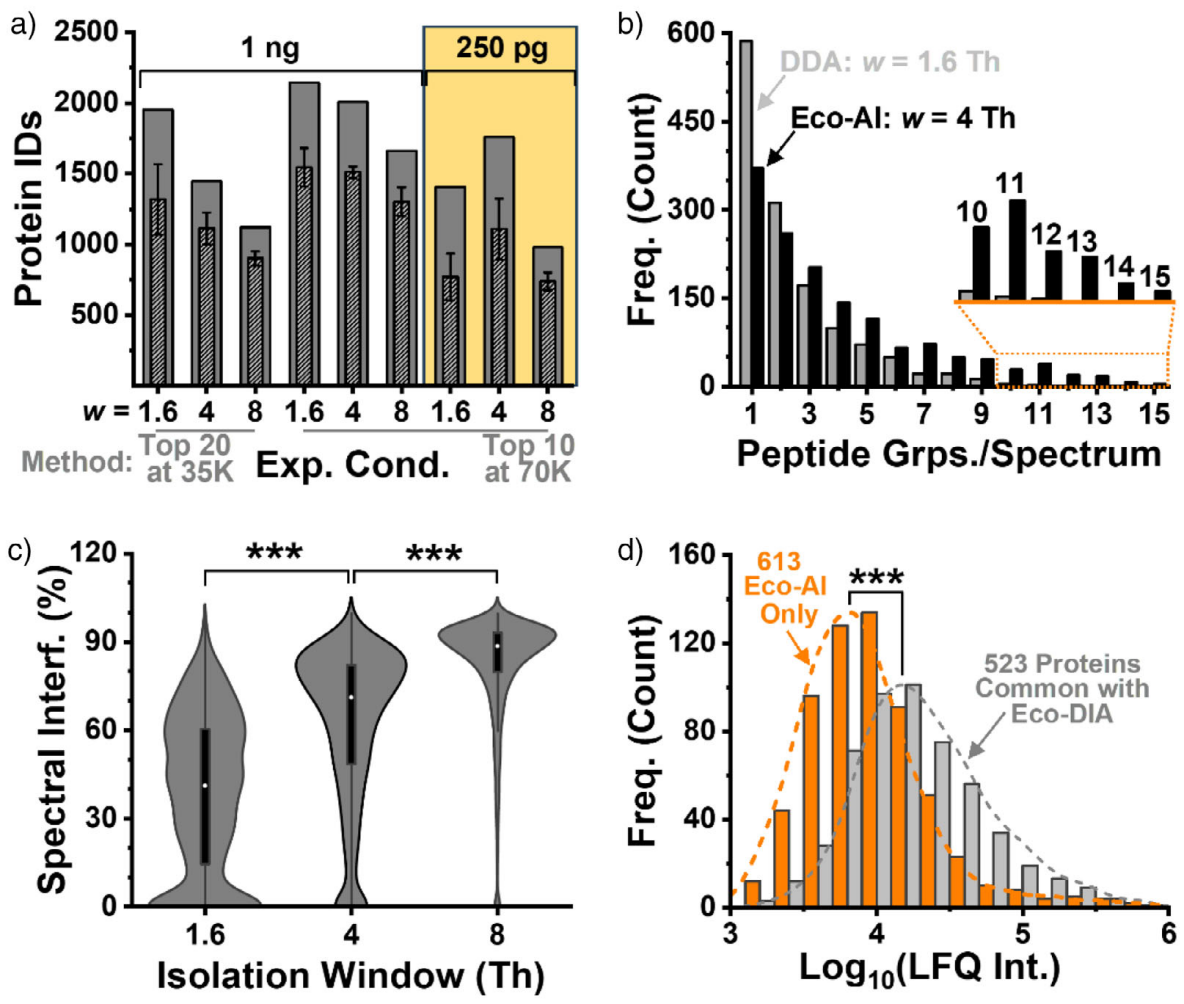
Configuration of empirical proteome depth using electrophoresis‐correlative data acquisition with AI‐aided data processing (Eco–AI). HeLa digest (1 ng and 250 pg) was analyzed under fixed MS cycle duration while varying key parameters: quadrupole isolation window (*w*), number of targeted precursor ions (Top 10 versus Top 20), and Orbitrap resolution (35 000 versus 70 000 FWHM). a) Eco–AI identified up to 1799 proteins in 15 min from ∼250 pg of digest—approximating the protein content of a single HeLa cell. b) The MS/MS spectra showed an increased peptide group depth (examples in Supporting Information), while c) wider quadrupole precursor ion isolation window widths exacerbated spectral interference. d) Label‐free quantification (LFQ intensity) confirmed superior sensitivity of Real‐Time Eco–AI over the recent (scheduled) Eco CE–DIA method. ***p < 0.001, Mann–Whitney U test.

We next examined how spectral resolution and isolation width influenced proteome depth at a fixed Orbitrap cycle time. From 1 ng (∼4–5 cells), narrow *w* and elevated resolution improved protein identifications (Figure 3a). The optimized configuration yielded 1483 ± 136 proteins in 15 min, totaling 2142 across triplicates (Table S2). This affords a theoretical throughput of 48 samples/day. At 250 pg (∼1 cell), a slightly wider 4 Th window proved optimal, enabling detection of up to 15 peptides per spectrum (Figure 3b). However, an 8 Th window elevated spectral interference, which in turn hindered protein identifications (Figure 3c, p < 0.001). The optimal *w* in our CE–MS study was broader than the typical ∼0.8–1.6 Th employed in classical DDA, yet narrower than recent efforts employing 15–25 Th widths in DIA^[^ ^19^, ^46^, ^47^, ^48^ ^]^ and 8–12 Th using DDA with WWA^[^ ^36^, ^37^ ^]^ in nanoLC. These results emphasize the need to tailor acquisition strategies to CE–MS.

Protein quantification performance was benchmarked against classical DDA,^[^ ^25^ ^]^ DIA,^[^ ^19^ ^]^ and our prior (scheduled) Eco–DIA,^[^ ^40^ ^]^ the closest reference technologies. On average, Real‐Time Eco–AI yielded 1106 ± 217 proteins per replicate (*n* = 4), totaling 1799 proteins without using match‐between‐runs (Table S3). This represents 4.5‐fold and 2.6‐fold improvements over DDA and DIA, respectively. Compared to Eco–DIA, the protein coverage doubled (Figure 3d). Notably, the 613 proteins uniquely quantified by Eco–AI dominated the lower abundance range (*p* = 1.54 × 10^−65^) based on the calculated label‐free index values used as a proxy.^[^ ^9^ ^]^ LFQ concentrations between 1 and 10 ng inputs correlated well (*ρ* = 0.92; Figure S4), and the coefficient of variation (CV) averaged ∼16.4% among the technical triplicates. These data confirm that Real‐Time Eco–AI enhances sensitivity while maintaining strong quantification reproducibility.

To support method transferability, we evaluated Real‐Time Eco–AI on the Orbitrap Fusion Lumos, importantly, without instrument‐specific optimization. Preliminary CE–MS runs on the tribrid platform yielded 1537, 1275, 1397, and 1542 proteins, averaging 1438 ± 127 proteins. This represents an approximate 30% increase in proteome coverage compared to the QE+ under comparable conditions, attributable to the Lumos's higher resolution (120 K versus 70 K), faster sequencing speed (4 versus 3 Hz), and improved ion transmission efficiency. Although unoptimized for this portion of the study, these results underscore both the compatibility of Real‐Time Eco–AI with advanced instrumentation and its potential to achieve even deeper coverage when leveraged on next‐generation mass spectrometers.

**Figure 4:**
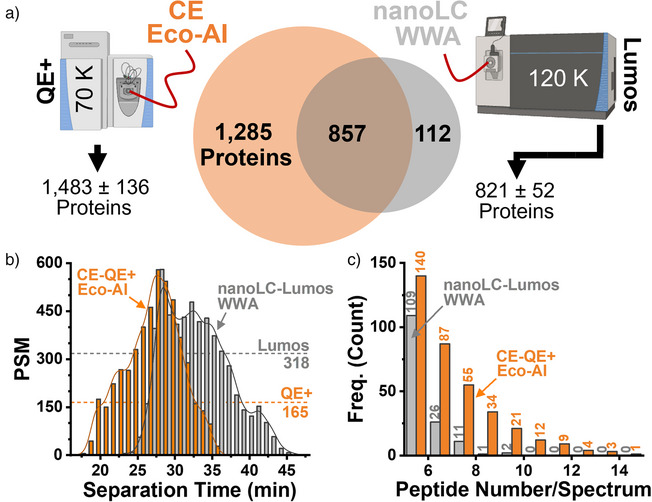
Benchmarking Real‐Time electrophoresis‐correlative (Eco) acquisition with AI‐aided processing against a modern nanoLC–tribrid–WWA workflow. A 1 ng HeLa digest was analyzed by capillary electrophoresis (CE) on a legacy Orbitrap mass spectrometer (Q Exactive Plus, QE+), and compared to nanoLC–MS on a tribrid Orbitrap (Fusion Lumos, Thermo) with higher ion transmission, faster scan rate (maximum 15 versus 13 Hz), and greater resolution (tested: QE+ at 70 000 FWHM and Fusion Lumos at 120 000 FWHM). a) Despite lower hardware specifications, Real‐Time Eco–AI yielded a marked sensitivity gain, validated also against independently obtained data (see Supporting Information). b) Peptide spectral match (PSM) rates reached levels comparable to the modern tribrid system. c) This gain resulted from richer tandem MS spectra acquired with Real‐Time Eco–AI compared to nanoLC‐WWA.

We further tested the Lumos architecture, optimized for nanoLC using the WWA modality (Figure S5). The platform identified 969 proteins from 1 ng in 5 replicates (median CV = 17.3%; Table S4). Real‐Time Eco–AI measured 2142 proteins in 3 replicates using a comparable ∼15‐min effective separation window. Most nanoLC–Lumos‐identified proteins were also detected by CE (Figure 4a), and QE+’s slower scan rate (165 MS^2^ min^−1^ versus 318 from nanoLC–Lumos) was compensated by CHIMERYS, achieving comparable PSM rates (<600 PSMs min^−1^, Figure 4b). Intriguingly, these rates rival recent CE–MS and nanoLC–MS studies^[^ ^18^, ^22^, ^49^ ^]^ using 2–7× longer separations and even faster and more sensitive Orbitraps, specifically the Exploris employing CHIMERYS (Figure S6). On a per‐scan basis, Eco–AI yielded significantly more peptides (Figure 4c), highlighting that legacy instruments can deliver state‐of‐the‐art single‐cell performance when paired with Real‐Time Eco–AI.

### Single‐Cell Differentiation Between D11 and V11 Lineages in the Embryo

**Figure 5:**
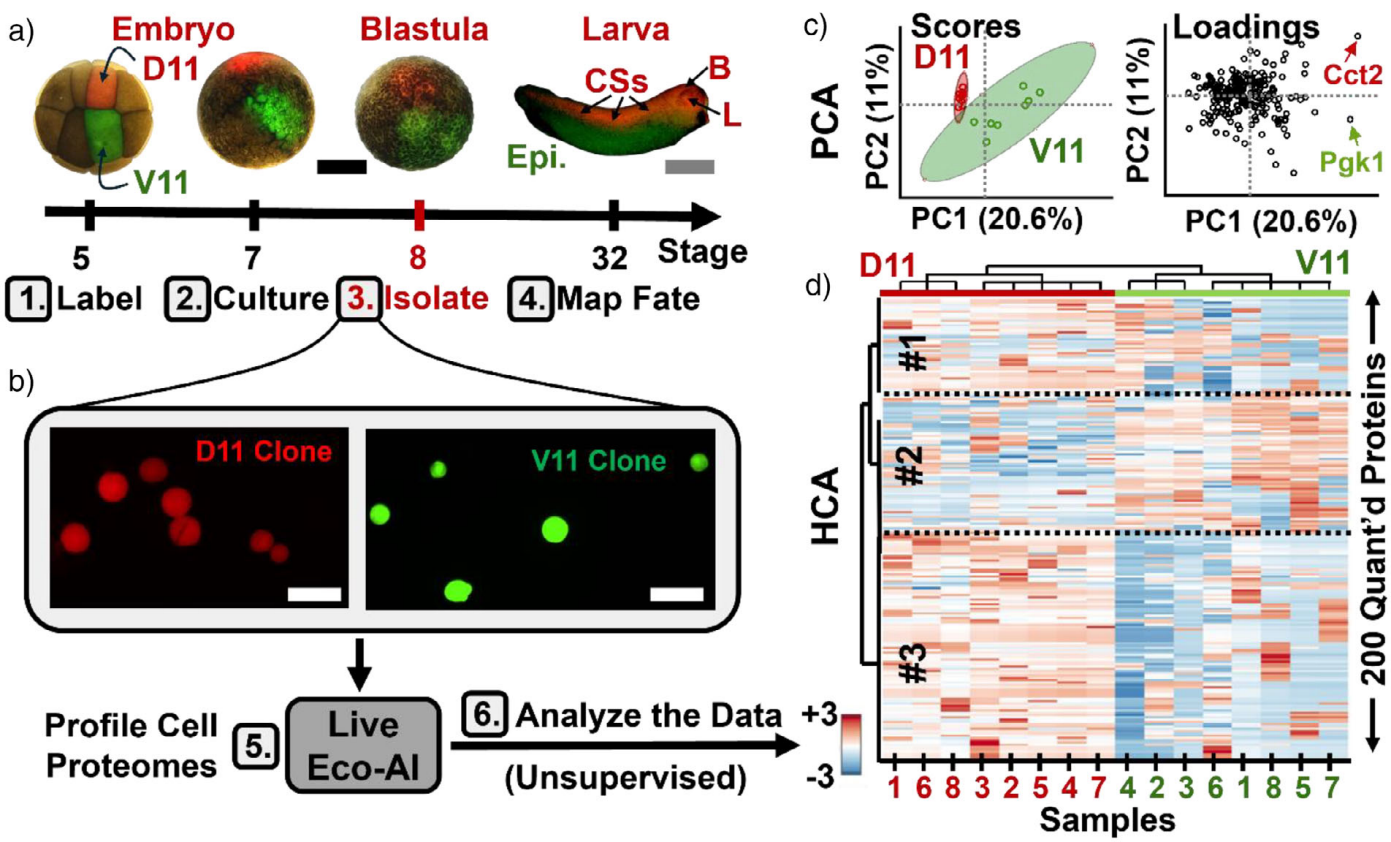
Proteome profiling of single cells undergoing differentiation in *Xenopus laevis* embryos. a) Dorsal‐animal (D11) and ventral‐animal (V11) blastomeres (*n* = 8) were microinjected with red and green fluorescent dyes at the 16‐cell stage; these lineages give rise to neural (central somites, CSs) and epidermal (Epi.) tissues in the larva, respectively. b) Descendant cells (∼50–75 µm) were isolated at the blastula stage using a micropipette under fluorescence guidance. ∼500 pg of proteome digest (∼2% of total cellular protein) was analyzed using the Real‐Time electrophoresis‐correlative AI workflow. c) Principal component analysis (PCA) of the cell proteomes revealed systematic differences among sample types (scores and loadings plots), driven by varying protein expression levels. Representative proteins are labeled. d) Hierarchical cluster analysis (HCA, z‐score scale) of the top 200 proteins grouped them into three major abundance profiles (#1–3), supporting cell‐type‐specific proteome remodeling. Scale bars: 500 µm (black), 2 mm (gray), 100 µm (white).

We applied Real‐Time Eco–AI on the QE+ mass spectrometer to profile proteome remodeling during early cell differentiation in *X. laevis*. Specifically, we compared the D11 and V11 blastomeres, which give rise to neural and epidermal tissues, respectively^[^ ^50^ ^]^ (Figure 1). Fluorescent lineage labeling of D11 (red) and V11 (green) in 16‐cell (Nieuwkoop Faber stage 5) embryos enabled fate tracing, which confirmed expected tissue outcomes at stage 32 (Figure 5a). By mid‐blastula (stage 8), the ∼250‐µm‐diameter blastomeres shrink to ∼50–75 µm, yielding ∼2.5 ng yolk‐free proteome (∼150 pL cytoplasm), atop an ∼90% yolk background.^[^ ^10^, ^51^ ^]^ We dissociated the fluorescence‐labeled tissues per established protocols,^[^ ^52^ ^]^ and manually isolated *n* = 8 D11 and *n* = 8 V11 descendant cells using micropipettes. The blastomeres were deposited on a microscope slide for phenotyping using optical fluorescence (Figure 5b). All the cells in this study were sourced from a single clutch of embryos from the same single pair of mother and father to minimize biological variability. To approximate sensitivity to mammalian cells, only ∼500 pg (containing ∼50 pg of total yolk‐free) proteome was analyzed per cell.

Real‐Time Eco–AI identified 1524 proteins cumulatively among the D11 and V11 blastomeres (Table S5). This reflects a ∼10‐fold sensitivity improvement and 2–4× higher throughput compared to custom CE platforms (reporting ∼800–1700 proteins from ∼5–10 ng inputs)^[^ ^19^, ^23^, ^25^, ^29^, ^53^ ^]^ and an ∼80‐fold sensitivity enhancement over recent nanoLC studies (644–1650 proteins from 40 ng to 2 µg).^[^ ^13^, ^54^ ^]^ Annotation via PantherDB 18^[^ ^55^ ^]^ showed that over 1200 proteins participated in binding (456 proteins), catalytic (489), structural (107), or transporter (34) functions. Many were involved in metabolism (606), stimulus response (112), and intracellular localization (128) (Figure S7). Compared to earlier DIA‐based CE–MS from precursor blastomeres on the same CE–MS platform,^[^ ^19^ ^]^ Real‐Time Eco–AI delivered significantly higher sensitivity (*p* = 5.6 × 10^−15^, Figure S8).

We next examined whether proteomic differences between dorsal and ventral lineages could be detected at the single‐cell level. Figure 5c shows a principal component analysis (PCA) based on median‐normalized, log₁₀‐transformed LFQ intensities. The top PCs described 20.6%, 11%, and 10% of variance in the dataset. The first two PCs separated the samples into two distinct clusters (scores plot). Upon revealing the identity of the cells, we learned that these groups corresponded to the dorsal versus ventral lineages. Notably, the data corresponding to the D11 (neural‐fated) cells showed tighter clustering, while the V11 (epidermal‐fated) were more dispersed, consistent with known morphogenetic behaviors: convergent extension for neural development versus epithelial dispersion for epidermis formation (Figure 5a). Proteins with comparable abundance among the cell types populated the origin. The PCA loadings plot revealed multiple proteins driving the separation. For example, Cct2 (PC1 = 0.25, PC2 = 0.21, *p* = 0.01) was enriched in D11 cells, while Pgk1 (PC1 = 0.24, PC2 = −0.08, *p* = 0.01) was elevated in V11 descendants.

Significance was confirmed using statistical models. Hierarchical clustering of the 200 most differential proteins also distinguished the two groups (Figure 5d; close‐up in Figure S9). Orthogonal to the PCA results (Figure 5c), this unsupervised method also revealed dissimilar sample types. The two groups corresponded to the D11 versus V11 lineages, as we learned on revealing the identity of the samples at this stage of the data analysis. Clustering resolved three major protein groups: #1 and #3 enriched in D11, and #2 enriched in V11. Examples include Cndp2 and Cct2 (cluster #1, D11) versus Pgk1 and Grhpr.2 (cluster #2, V11). Notably, several proteins matched earlier findings in 16‐cell embryos^[^ ^12^ ^]^: Rpl31, Calm1, and Rpl29 were D11‐enriched, while Ckb was enriched in V11.

To explore functional relevance, we queried *Xenopus* gene expression via Xenbase.^[^ ^56^ ^]^ Genes such as *cct3* and *eif5a* were enriched in dorsal/neural lineages. In situ hybridization confirmed *cct3* expression in neural tube, CNS, brain, and eye.^[^ ^57^ ^]^ *Eif5a* is a known effector of neuronal outgrowth.^[^ ^58^ ^]^ Conversely, *hba‐l5* (alpha globin larval‐5) was enriched in V11 progeny, consistent with its role in ventral blood island,^[^ ^59^ ^]^ non‐neural ectoderm, and pronephros formation,^[^ ^50^, ^60^ ^]^ the hallmark features of the V11 fate. Although functional roles for several observed proteins remain untested in this report, our data establish Real‐Time Eco–AI as a powerful tool for detecting molecular changes during early cell fate decisions.

To assess the biological breadth of this technology, we annotated the differentially expressed proteins using the PANTHER database 18.0 (Figure S7). Many proteins from all three clusters were involved in core biosynthetic functions, including over 30 ribosomal proteins, translation factors (e.g., Eif4ai, Eef1b, and Eef2), and chaperones (e.g., Cct2 and Cct6a). Various energy metabolism pathways were also prominent, including electron transport (cluster #2: Cox7a2 and Ndufv1), ATP synthesis (cluster #3: Atp5f1a, Atp5f1c, Atp5pb, Atp5pd, and Atp5po), mitochondrial transport (Vdac3), glycolysis (cluster #2: Pgk1 and Gapdh), and the Krebs cycle (cluster #3: Mdh1 and Mdh2 catalyzing malate–oxaloacetate conversion). These data underscore the importance of energy currency to fuel cell division.

## Conclusion

We developed Real‐Time Eco–AI as a cost‐effective strategy for single‐cell proteomics that complements the performance of modern, high‐end platforms. By custom‐building a CE‐based separation platform to Eco‐sort peptide ions by *m*/*z* and integrating real‐time acquisition logic, we enabled efficient MS^2^ sequencing on a legacy orbitrap mass spectrometer (QE+). Despite the instrument's slower acquisition speed and resolution and reduced ion collection/transfer efficacy, Real‐Time Eco–AI achieved deep proteome coverage, identifying 1799 proteins from a single HeLa‐cell‐equivalent (∼250 pg) digest within 15 min of effective separation, raising the potential for a 48 samples‐per‐day throughput. The observed peptide identification rates were on par with the modern‐generation orbitrap altervatived (Fusion Lumos and Exploris tested).

Applying to *Xenopus laevis* embryonic cells, Real‐Time Eco–AI identified 1524 proteins from ∼500 pg (50 pg of yolk‐free) of proteome during the 10 min of effective CE–MS analysis. This corresponds to a theoretical throughput of 72 samples per day. These data revealed reproducible proteomic differences between dorsal (D11) and ventral (V11) lineages at the blastula stage, consistent with established embryonic asymmetries and confirming biological validity. To our knowledge, this represents the deepest single‐cell proteome coverage reported for mid‐blastula *X. laevis* embryos, achieved using only a small fraction of the available cellular material. By enhancing sensitivity while minimizing instrument cost, Real‐Time Eco–AI expands access to high‐resolution single‐cell proteomics and positions CE–MS as a complementary alternative to state‐of‐the‐art nanoLC–MS platforms.

By enhancing sensitivity while significantly lowering instrumentation cost, we anticipate Real‐Time Eco–AI to broaden access to high‐resolution single‐cell proteomics through CE–MS. This technology has matured into a viable, scalable, and complementary alternative to advanced nanoLC–MS platforms for trace‐sensitive, including single‐cell 'omics studies. Although demonstrated here on a custom‐built CE system, our accumulated experience shows that the workflow is fully compatible with commercial CE hardware and can be implemented using open‐source or vendor‐neutral control software, ensuring seamless integration with existing laboratory infrastructure and facilitating widespread transferability.

## Author Contributions

P.N. conceptualized the study. P.N. and B.S. designed the research objectives. F.Z. labeled, dissociated, and imaged the single blastomeres. B.S. developed the technology, processed the blastomeres, and measured the blastomere proteomes. F.Z. assisted during the measurements. B.S. and P.N. analyzed the data and interpreted the results. B.S. prepared the draft report. P.N. revised and finalized the manuscript. P.N. acquired the funding. All the authors commented on the manuscript.

## Conflict of Interests

The authors declare no conflict of interest.

## Supporting information



Supporting Information

Supporting Information

## Data Availability

The data that support the findings of this study are openly available in the Proteomics Identification Database (PRIDE), https://www.ebi.ac.uk/pride/, under the reference number 62702.
