# Supplementary material for: Real‐Time Eco–AI, Electrophoresis‐Correlative Data‐Dependent Acquisition with AI‐Based Data Processing Broadens Access to Single‐Cell Mass Spectrometry Proteomics
Source: Angew Chem Int Ed Engl. 2025 Aug 23;64(45):e202510692. doi: 10.1002/anie.202510692 (PMC12582007; doi:10.1002/anie.202510692)
Supplement: Supplementary file 1 — Supporting Information [file ANIE-64-e202510692-s001.pdf]

Supporting Information for

Real-Time Eco–AI, Electrophoresis-Correlative Data-Dependent Acquisition with AI-Based Data Processing Broadens Access to Single-Cell Mass Spectrometry Proteomics

Bowen Shen[1], Fei Zhou[1], and Peter Nemes[1]* (*nemes@umd.edu)

[1]Department of Chemistry & Biochemistry, University of Maryland, College Park, MD 20742

Table of Contents

SI METHODS 2
Materials 2
Solutions and Media 2
Animal Care and Embryology 2
Single-Cell Isolation and Proteome Processing 2
CE-ESI-MS Analysis 3
Ionization 3
Detection 3
NanoLC-ESI-MS Analysis 3
Data Analysis 4
Quantification 4
Models for CE 4
Scientific Rigor 4
Safety 4
Data Availability 5
SI FIGURES 6
Figure S1. Peptide separation vs. protein identification. 6
Figure S2. Representative annotation of peptide spectral matches 8
Figure S3. Corroborative analysis of the peptide identifications 9
Figure S4. Cross-correlation analysis of proteome quantification 10
Figure S5. Configuration of experimentation conditions 11
Figure S6. Performance benchmarking 12
Figure S7. Interpretation of canonical knowledge 13
Figure S8. Comparison of Real-Time Eco–AI sensitivity against the DIA–Eco reference 14
Figure S9. Close-up of the HCA-heat map 15
SI REFERENCES 16

SI METHODS

Materials. HPLC-grade solvents and chemicals, including acetic acid (AcOH), acetonitrile (ACN), formic acid (FA), and methanol (MeOH) were obtained from Thermo Fisher Scientific. Ammonium bicarbonate (AmBic) was purchased from Avantor (Center Valley, PA). The HeLa proteome digest standard (part no. 88329, Pierce, Rockford, IL) and N-dodecyl-β-D-maltoside (DDM, part no. 89903, Rockford, IL) were provided by Thermo Fisher Scientific. The fused silica capillaries were purchased from Polymicro Technologies (40/105 µm inner/outer diameter, part no. 1068150596, Phoenix, AZ) for CE analysis. The CE-nanoESI emitters were fabricated from borosilicate glass capillaries (0.75/1.00 mm inner/outer diameter, part no. B100-75-10, Sutter Instrument, Novato, CA). Proteome digestion was performed with MS-grade Trypsin Platinum (part no. VA900A, Promega, Madison, WI). To minimize analyte loss during sample preparation, all samples were processed in 0.5-mL LoBind vials (Eppendorf, cat no. 022431064, Enfield, CT).

Solutions and Media. The CE background electrolyte (BGE) was prepared to contain 1 M FA in 25% (v/v) ACN. The CE-ESI sheath solution comprised 0.5% (v/v) AcOH in 10% (v/v) MeOH. The HeLa proteome digest or single cell samples were dissolved in the sample solvent prepared with 0.05% (v/v) FA in 75% (v/v) ACN. The embryo culture media was prepared with 3% Ficoll in 100% Steinberg's solution following standard protocols.[1-2] For fluorescent labeling, the fluorescent dextran solution was prepared with 0.5% (v/v) green dextran in DEPC-treated water. For tissue dissociation, the Newport buffer was prepared as described elsewhere.[3-4] The proteome lysis solution consisted of 0.2% DDM in 50 mM AmBic.

Animal Care and Embryology. Sexually mature *X. laevis* frogs were supplied by Xenopus1 (Dexter, MI) or Nasco (Fort Atkinson, WI). All procedures related to the humane care and management of *X. laevis* were authorized by the Institutional Animal Care and Use Committee at the University of Maryland, College Park (approval no. R-FEB-21-07 or R-FEB-24-05). To capture natural biological variability in the data, the embryos were obtained through the natural mating of one pair of parents. Two-cell embryos presenting stereotypical pigmentation[5] were cultured to the 16-cell stage (Nieuwkoop-Faber[6], NF stage 5), where the cell types were readily identifiable based on pigmentation, size, and location in reference to reproducible cell-fate maps.[7] To trace tissue lineages, the left dorsal-animal midline (termed D11) or the ventral-animal midline (termed V11) cell of the 16-cell embryo was injected with 1 nL (0.5% v/v) of dextran (Alexa Fluor 488, 10,000 g/mol formula weight, anionic, Thermo Fischer). These fluorescently labeled embryos were cultured in 3% Ficoll in 100% Steinberg's solution at room temperature to the mid blastula stage (NF stage 8).

Single-Cell Isolation and Proteome Processing. Using sharpened forceps, the vitelline membrane ensheathing the embryo was carefully dissected away to isolate the fluorescence-labeled tissue under a fluorescence stereomicroscope. The biopsy was gently transferred to a glass vial containing Newport buffer, followed by gentle nutation to obtain a cell suspension (24 RPM speed for 10 min, ambient temperature, series no. I2CF61041100, Fisher Scientific). Using a 1 µL pipettor, the cells were carefully transferred onto a culture plate, then swiftly rinsed (in <1 min) with HPLC water to wash off salts and the complex media surrounding the cells. The resulting cells appeared intact based on the emission that the GD label produced in the D11 or V11 cells under a fluorescent stereomicroscope with FITC filter (Nikon SMZ18 with the excitation wavelength at 488 nm). Each cell was lysed by addition of 1 µL of the *proteome lysis buffer* at room temperature for 10 min. The single-cell proteomes were denatured by heating to

60 °C for 15 min, before digestion at 40 °C for 5 h by the addition of 1 µL of 0.1 µg/µL trypsin platinum in 50 mM AmBic. The classical steps of proteome reduction and alkylation were omitted to alleviate sample loss and simplify the workflow.[8] To compensate for liquid evaporation, the Eppendorf vials were kept tightly capped, and the contents of the vial were combined via intermittent centrifugation, every ~15 min. The single-cell samples were dried at room temperature, then stored at –80 °C until analysis.

CE-ESI-MS Analysis. *Separation.* The single-cell proteomes were analyzed on the same CE-nanoESI platform following the same protocols that we recently described in detail.[8-10] In this study, ~1 ng or ~250 pg of the HeLa proteome digest was electrophoresed at +250 V/cm electrical field strength in a background electrolyte (BGE)-filled 100-cm-long capillary (vs. Earth-grounded capillary outlet).

Ionization. The capillary outlet was connected to an electrokinetically pumped (+500–800 V) sheath-flow CE-nanoESI interface, built following a previous design[11] and operated in the cone-jet regime[12] for maximal ionization[13]. A stable Taylor cone was visualized under a long-working-distance objective microscope (Mitutoyo Plan Apo, Edmund Optics, Barrington, NJ) with a CCD camera (EO-2018C, Edmund Optics). This interface was affixed to a 3-axis translation stage to position the tip of the CE-ESI source ~1 mm from the grounded orifice of a mass spectrometer. The generated peptide ions were measured on a quadrupole–orbitrap tandem high-resolution mass spectrometer (QE+, Thermo Fisher Scientific), under control by data-dependent acquisition (DDA).

Detection. Two Top-N methods were devised for CE-MS Eco–AI. In both approaches, survey (MS^1) scans were obtained with the following parameters: Orbitrap (OT) mass resolution, 70,000 full width at half maximum (FWHM, at m/z 200); scan range, *m/z* 350–1,600; C-trap maximum injection time, 240 ms; AGC target for MS^1 and MS^2, 3×10^6 counts (both); charge states triggering MS^2, +2, +3, and +4. During the “Top-20” method, the 20 most abundant ("top") precursor ions were fragmented, the fragments C-trapped in <110 ms, and analyzed at 35,000 FHWM resolution in the OT. In contrast, the Top-10 method targeted fewer, specifically 10 “top” precursor ions, thus enabling longer times for C-trapping (<240 ms) and higher-resolution detection (70,000 FHWM). In both data acquisition modalities, the ion signals were dissociated in nitrogen collision gas at 28% normalized collision energy (NCE) in the higher-energy collisional dissociation (HCD) cell. The isolation widths of 1.6, 4, and 8 Th were assessed for each method. The *X. laevis* single-cell proteomes were measured using the Top-10 approach, with a 4 Th isolation window.

NanoLC-ESI-MS Analysis. The HeLa proteome digest was reconstituted in LC-MS grade water containing 0.1% (v/v) FA. One nanogram of the HeLa proteome digest was trapped (C18 trap column, 100 µm i.d., 5 µm particle with 100 Å pores, 2 cm length, Acclaim PepMap 100, Thermo) at 5 µL/min for 5 min. This proteome digest was separated on a 200 cm µPAC HPLC column (S/N 1100462, Thermo) at a 600 nL/min flow rate. A nanoflow liquid chromatograph (Dionex Ultimate 3000 RSLC, Thermo) provided a 60 min gradient of Buffer B (100% ACN, 0.1% v/v FA) as follows: from 1% to 22.5% over 22.5 min, then from 22.5% to 40% over 7.5 min, then from 40% to 95% over 5 min, and held for 95% for 10 min, then decreased to 1% in 3 min and equilibrated at 1% for 12 min. The peptides were charged in an electrospray source of an electrified (+2,300 V vs. Earth-ground) stainless-steel emitter (part no. ES542, Thermo Fisher). The peptide ions were detected on a quadrupole-ion trap-orbitrap tribrid-geometry high-resolution mass spectrometer (Orbitrap Fusion Lumos, Thermo). Peptide signals were surveyed

(MS^1) between *m/z* 380–1,600 at 120,000 FWHM in the Orbitrap analyzer every 3 s. The threshold was set to 2.0×10^4 counts, with AGC normalized up to 250% using auto maximum injection time and a 30 s dynamic exclusion. A DDA method was programmed to isolate the precursor ions within windows of *m/z* 1.6, 4, 8, 12, or 20 Th, followed by fragmentation in the HCD cell at 30% NCE. The resulting MS^2 spectra were recorded at 60,000 FWHM.

Data Analysis. *Protein Identification.* The Eco–AI MS data were analyzed in Proteome Discoverer 3.0 (Thermo Fisher Scientific), executing CHIMERYS (Prediction model: inferys_2.1_fragmentation) for protein identification and quantification. The MS^2 acquisition was extracted from Proteome Discoverer 3.0 (Thermo Fisher Scientific), executing Sequest HT for the conventional DDA analysis without AI. Protein identifications were searched against the HeLa Proteome (UP000005640, downloaded from UniProt in July 2023, containing 20,523 entries). The search parameters included static modification (cysteine carbamidomethylation), dynamic modifications (methionine oxidation), minimum and maximum peptide length (7 and 30 amino acids, resp.), maximal missed cleavage sites (2), fragment ion mass tolerance (20 ppm), and the search for common contaminants (enabled). Similarly, the MS data from experiments on the *X. laevis* were mapped against the *X. laevis* proteome (UP000694892, downloaded in April 2022, containing 42,596 entries), employing the same parameters for the HeLa. Peptide and protein identifications were based on at least 1 proteotypic peptide, filtered to <1% false discovery rate (FDR), computed vs a decoy proteome database enlisting the reversed proteome sequence. For a direct comparison between Eco–AI and DIA performed on CE-MS, protein identification and quantification were obtained with DIA-NN 1.9[14] with the settings as follows: minimal peptide length, 5; maximal peptide length, 35; maximum missed cleavage, 2; and precursor charge, 2–4. All the other parameters were set to default.

Quantification. The LFQ data were median normalized to compare identical proteome amounts, $\log_{10}$-transformed to reduce the quantitative dynamic range with MetaboAnalyst 6.0[15], and analyzed through statistical means in OriginPro 2020b (Origin Lab, Northampton, MA). The MFs were surveyed in MzMine 3.9[16] with the settings: m/z range, 380–1,200; noise level, 10^4 counts; scans, MS^1; minimum signal height, 10^4 counts; *m/z* tolerance, 0.05 Th or 20 ppm. For the non-parametric statistical test, the Mann-Whitney *U* test was performed with R to yield the exact p values unless it reached the limitation.

Models for CE. The electrophoretic mobilities were calculated following our previous work[17] using the following experimental setting: BGE pH, 2.3. The effective electrophoretic mobilities (μ_{eff}) were calculated from the empirical migration times as described elsewhere[18] considering the following settings: capillary length, 1.0 m; CE separation potential, 25,000 V. The theoretical μ_{eff} values were predicted based on the anticipated charge state and size of the peptides as developed elsewhere[19] considering the following experimental parameter: BGE pH, 2.3.

Scientific Rigor. Each HeLa proteome digest was analyzed in 3–5 technical replicates (the same sample measured multiple times). A total of n = 16 different blastomeres (biological replicates) were calculated from *X. laevis*. The cell type was identified based on various factors, including pigmentation, size, location, and the fluorescent emission visualized under a stereomicroscope. The biological replicates were analyzed randomly, and the mass spectrometer was calibrated and validated monthly to maintain a good performance.

Safety. Standard safety protocols were followed when handling all chemicals and biological samples. Careful precautions were taken during the manipulation of capillaries and ESI emitters

to mitigate potential puncture hazards. All electrically conductive components of the CE-HRMS platform were grounded or shielded from exposure within an enclosure with a safety interlock.

Data Availability. All the MS primary files and MS^2 spectral libraries and the HeLa and *Xenopus* proteomes were deposited in the Proteome Exchange Consortium via the PRIDE partner repository with the data set identifier PXD062702.

SI FIGURES

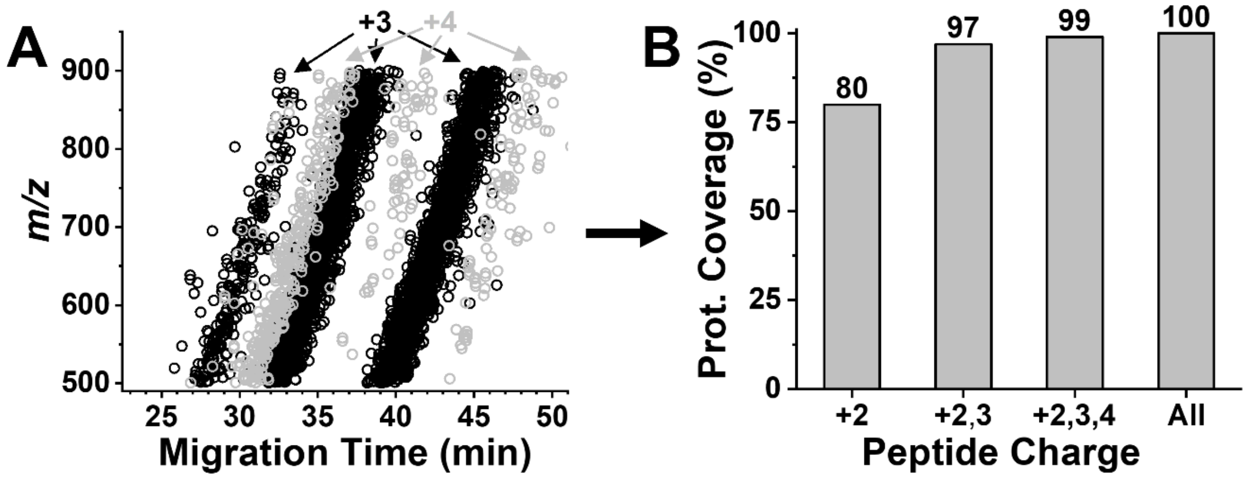

Figure S1. Peptide separation vs. protein identification. (A) Eco-sorting at the +3 and +4 charge states (protonated). **(B)** Cumulative coverage of the proteome over all the unknown and +2 or higher charge states measured. Accounting for ~80% of the identified proteome, +2 charge state was selected for results interpretation in this study.

<u>Example 1: 5 Peptides /MS² Spectrum</u> (MT, 18.5189 min; Precursor, *m/z* 471.2299)

Peptide #	Peptide sequence	Q Value
**Peptide 1**	**[R].GLVLDHGAR.[H]**	**3.00×10^{-4}**
**Peptide 2**	**[K].SHVDLFPK.[D]**	**3.00×10^{-4}**
**Peptide 3**	**[K].SIFLVAHR.[K]**	**3.00×10^{-4}**
**Peptide 4**	**[K].HELIEFR.[R]**	**3.00×10^{-4}**
**Peptide 5**	**[K].SDEGHPFR.[A]**	**1.70×10^{-3}**

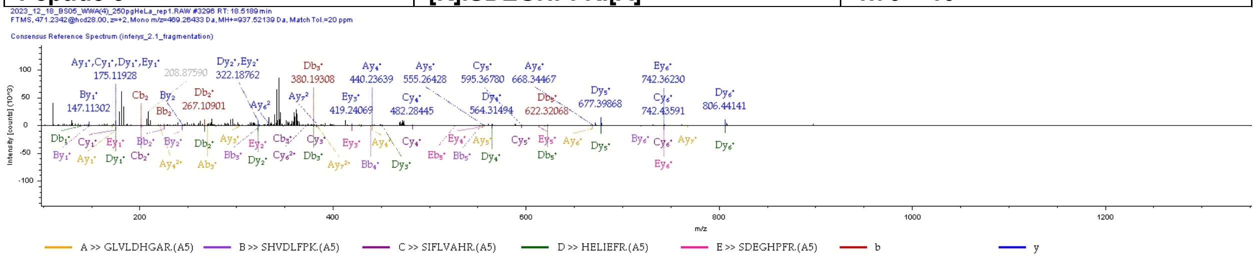

<u>Example 2: 10 Peptides/MS² Spectrum</u> (MT, 24.1379 min; Precursor, *m/z* 544.7870

Peptide #	Peptide Sequence	Q Value
**Peptide 1**	**[K].VELDNMPLR.[G]**	**3.00×10^{-4}**
**Peptide 2**	**[R].SLETENAGLR.[L]**	**3.00×10^{-4}**
**Peptide 3**	**[K].GLSQSALPYR.[R]**	**4.80×10^{-4}**
**Peptide 4**	**[K].SLNDLIEER.[Y]**	**1.50×10^{-3}**
**Peptide 5**	**[K].QFAEMYVAK.[F]**	**1.70×10^{-3}**
**Peptide 6**	**[R].LVSLYFDTK.[R]**	**3.10×10^{-3}**
**Peptide 7**	**[K].LLPDDPYEK.[A]**	**3.90×10^{-3}**
**Peptide 8**	**[K].SSEQILATLK.[G]**	**4.70×10^{-3}**
**Peptide 9**	**[K].NQAPPGLYTK.[T]**	**4.70×10^{-3}**
**Peptide 10**	**[K].TISPMVMDAK.[A]**	**5.60×10^{-3}**

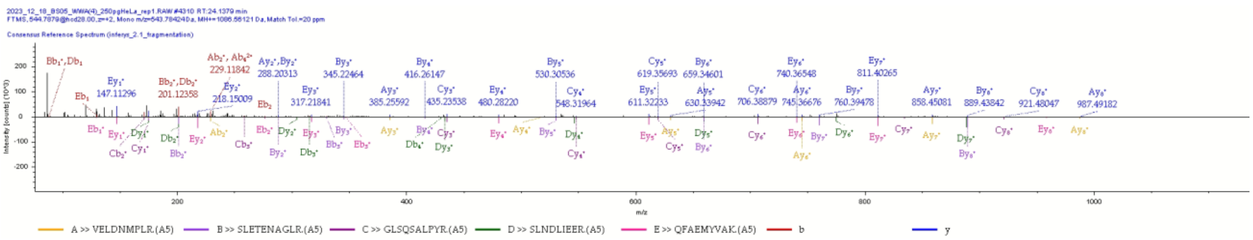

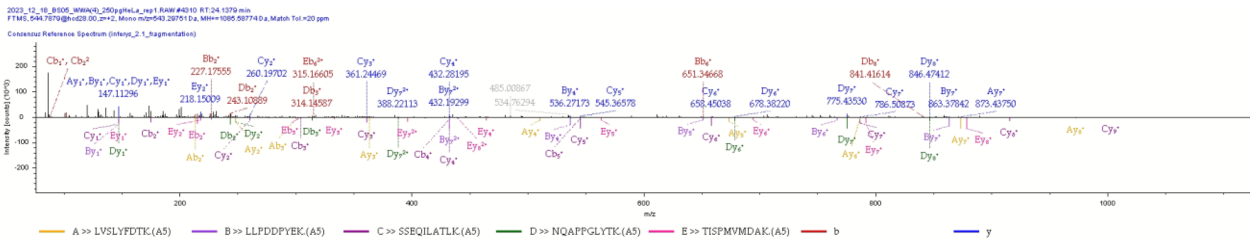

(Figure continued, next page)

(Figure continued from previous page)

<u>Example 3: 15 Peptides /MS^2 Spectrum</u> (MT, 26.3980 min; Precursor, *m/z* 714.8617)

Peptide #	Peptide Sequence	Q V
Peptide 1	[K].AFGPGLQGGSAGSPAR.[F]	3.00E-04
Peptide 2	[R].MGPAMGPALGAGIER.[M]	3.00E-04
Peptide 3	[R].SLYASSPGGVYATR.[S]	3.00E-04
Peptide 4	[R].EIADGLcLEVEGK.[M]	3.00E-04
Peptide 5	[R].VDQSAVGFEYQGK.[T]	3.00E-04
Peptide 6	[K].NVLIVEDIIDTGK.[T]	3.00E-04
Peptide 7	[K].GQEVETSVTYYR.[L]	3.00E-04
Peptide 8	[R].ITSPLMEPSSIEK.[I]	3.00E-04
Peptide 9	[R].LGSTVFVANLDYK.[V]	3.00E-04
Peptide 10	[R].VLSGDLGQLPTGIR.[D]	3.00E-04
Peptide 11	[R].GTIEILSDVQLIK.[T]	3.00E-04
Peptide 12	[K].LVGPEEALSPGEAR.[D]	3.00E-04
Peptide 13	[K].LSDLLAPISEQIK.[E]	4.80E-04
Peptide 14	[K].NWYLPAPEVSPR.[N]	4.60E-03
Peptide 15	[R].TPAIPTAVNLADSR.[T]	4.70E-03

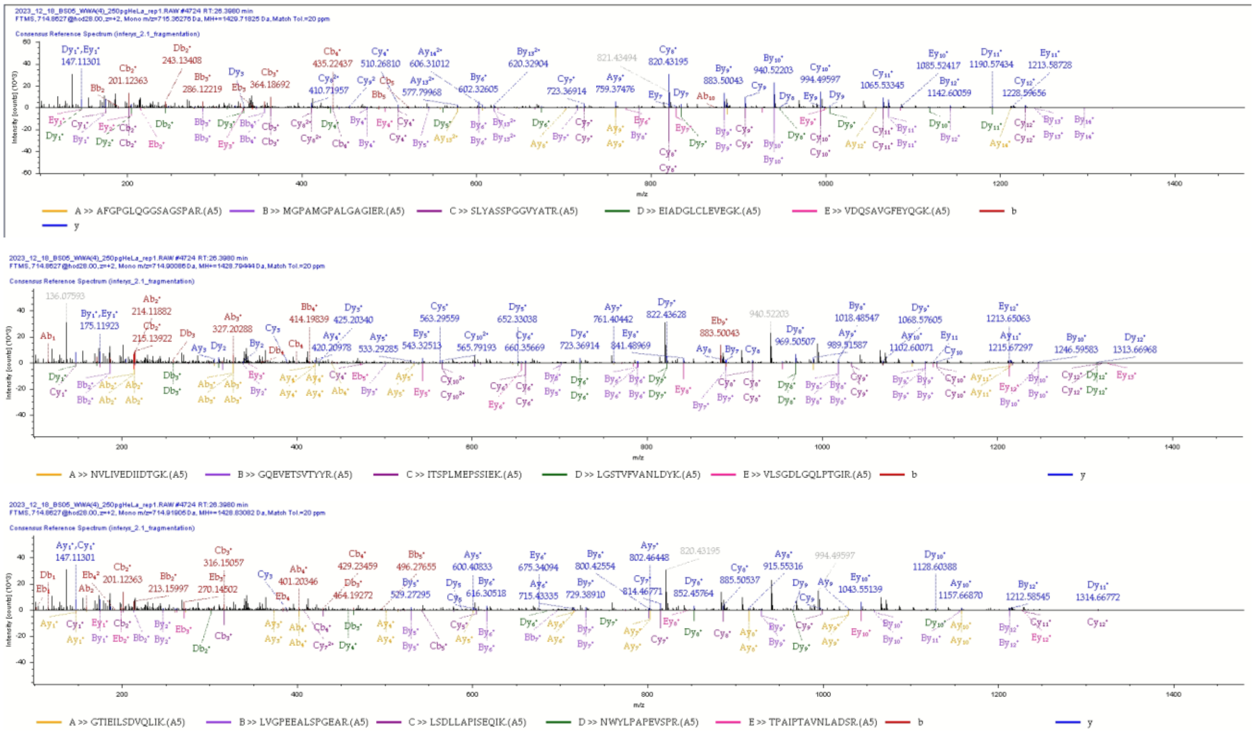

Figure S2. Representative annotation of peptide spectral matches (PSMs). The AI-assisted CHIMERYS search algorithm was used following the vendor's protocols. A Percolator Q value < 0.01 (1%) marks acceptable false discovery rates. The mirror plot of the experimental spectrum (top) and PSMs identified by CHIMERYS (bottom) at 1% FDR. Each plot shows 5 peptides matched.

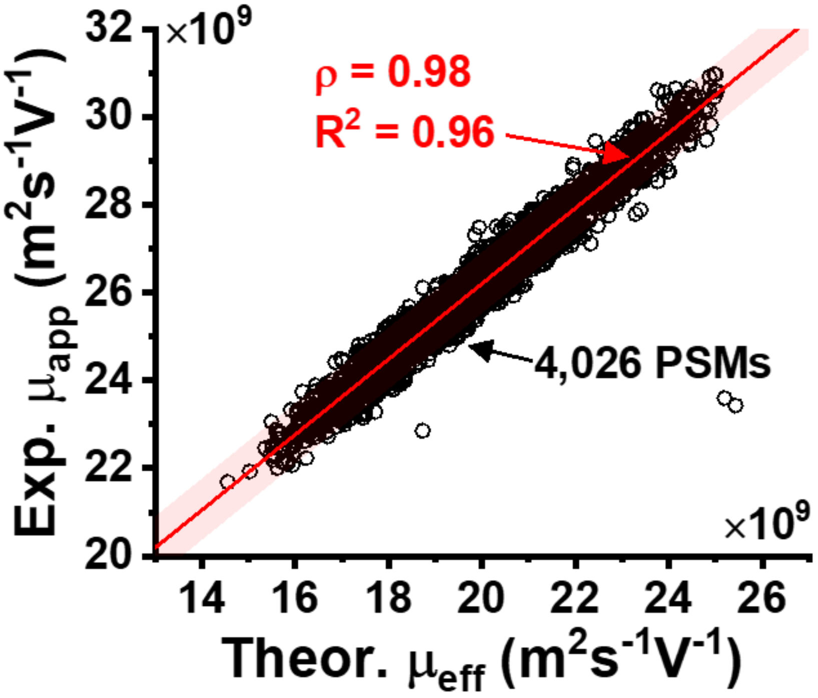

Figure S3. Corroborative analysis of the peptide identifications. Empirical (apparent, μ_{app}) and theoretical (effective, μ_{eff}) electrophoretic mobilities were highly correlated. Shown is a Pearson correlation analysis for the 4,026 PSMs (+2 charge state) that were extracted from the middle trend line (**Fig. 2F**) using CHIMERYS. Low false discovery rates (<1%) and high correlation with the theoretical μ_{eff} supported the accuracy of the peptide identifications.

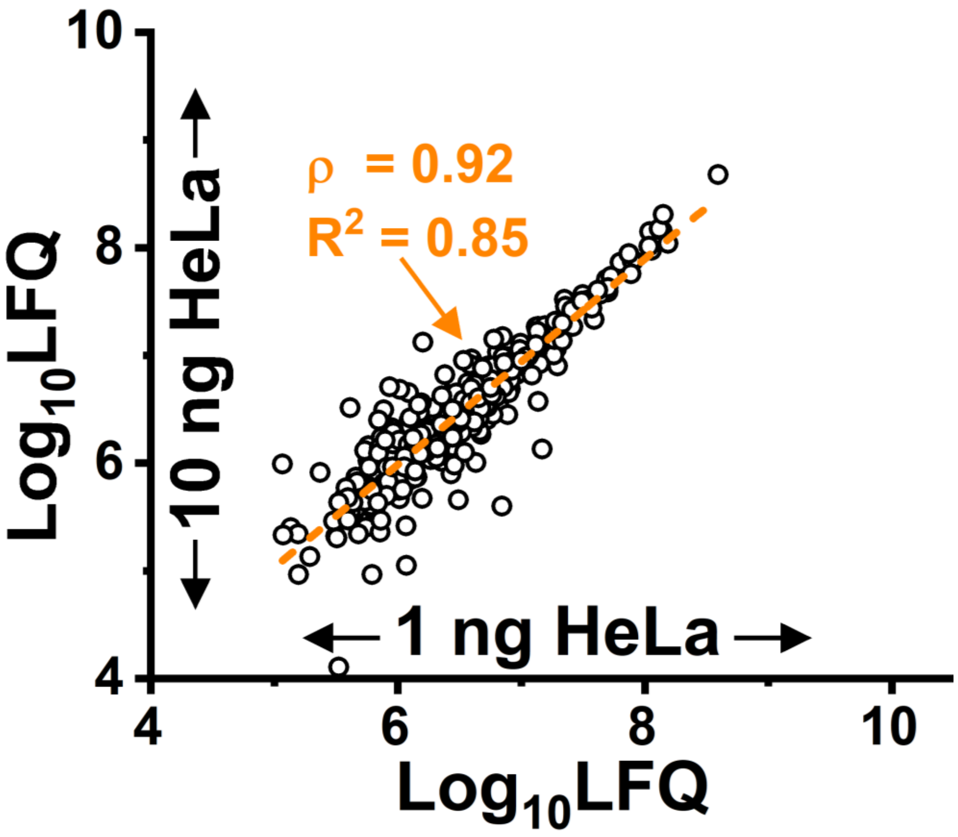

Figure S4. Cross-correlation analysis of proteome quantification. 1 ng and 10 ng of the HeLa proteome digest were measured. The protein concentrations were estimated based on LFQ. The high Pearson correlation moments (ρ) and linear regression coefficients (R^2) calculated between the LFQ concentrations revealed robust quantification using Real-Time Eco–AI.

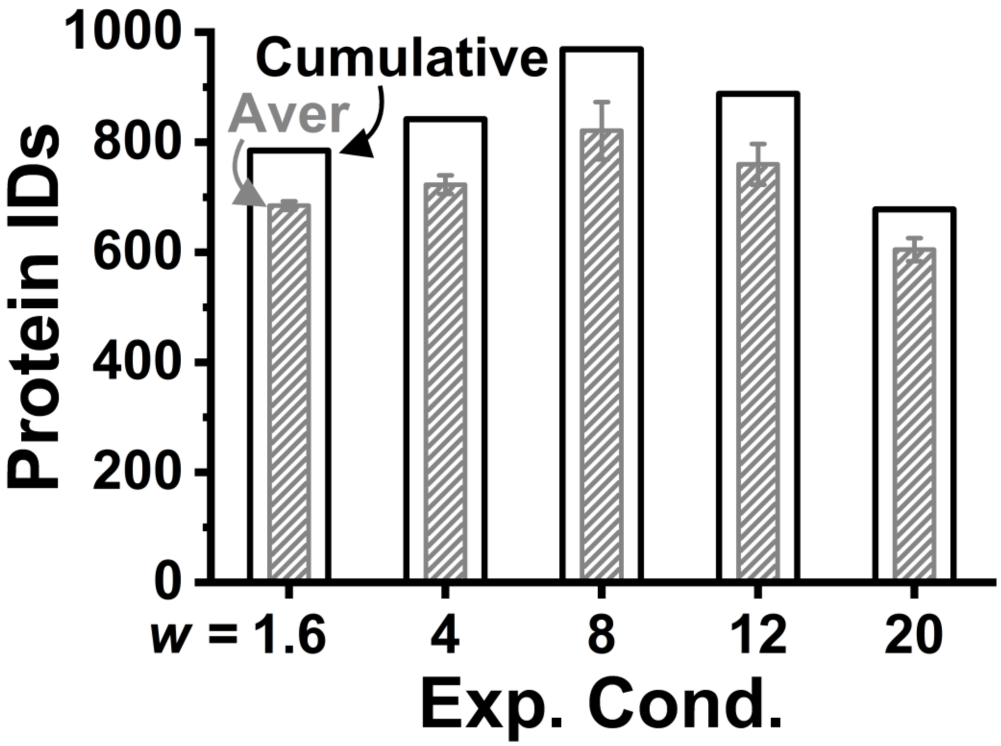

Figure S5. Configuration of experimentation conditions (Exp. Cond.) to improve proteome sensitivity using nanoLC with the Orbitrap Fusion Lumos mass spectrometer. The wide-window acquisition (WWA) method was tested with different quadrupole precursor m/z isolation windows (*w*). 1 ng of the HeLa proteome digest was separated using a 30-min active reversed-phase gradient. The separated peptides were ionized in a nano-flow electrospray ionization source and detected on a modern tribrid mass spectrometer (Fusion Lumos, Thermo). Average and cumulative protein identifications are compared among n = 4–5 technical replicates.

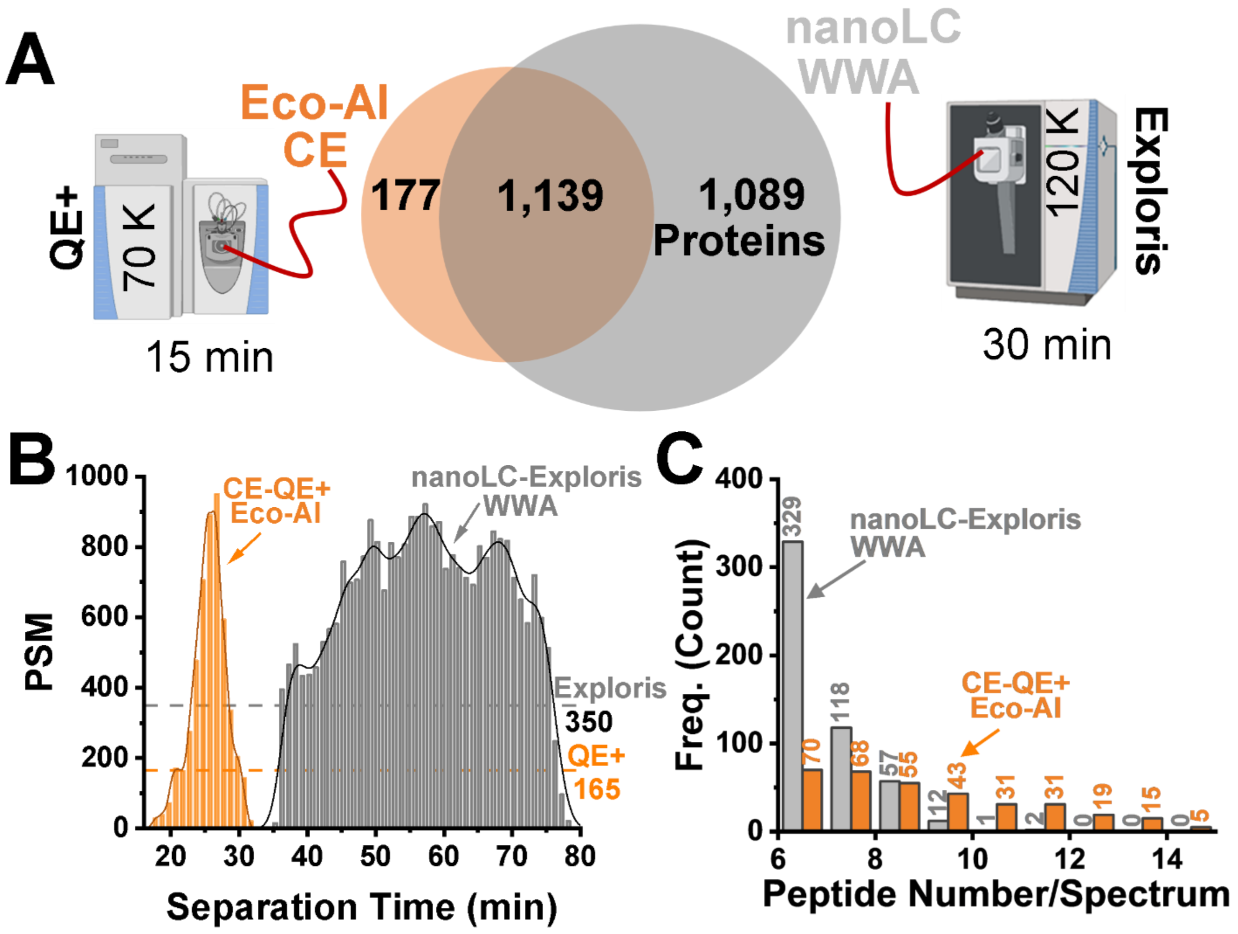

Figure S6. Performance benchmarking Real-Time Eco–AI on the Q Exactive Plus (QE+) vs the modern nanoLC Exploris MS reference. Single-cell-equivalent HeLa proteome digests were measured using both platforms (250–200 pg). The nanoLC-MS results were obtained on an Exploris 480 employing wide window acquisition (WWA) in an independent study published elsewhere.[20] **(A)** Although the final proteome depth was higher using the Exploris with elevated sensitivity, resolution, and speed, **(B)** the experimental rate of peptide sequencing was comparably high between CE-MS and nanoLC-MS. **(C)** The Real-Time Eco–AI on the QE+ could extract more peptides per MS^2 spectrum despite one-third of the scan rate and the separation time used in the nanoLC-WWA reference. These results demonstrate quasi-matching performance among the technologies.

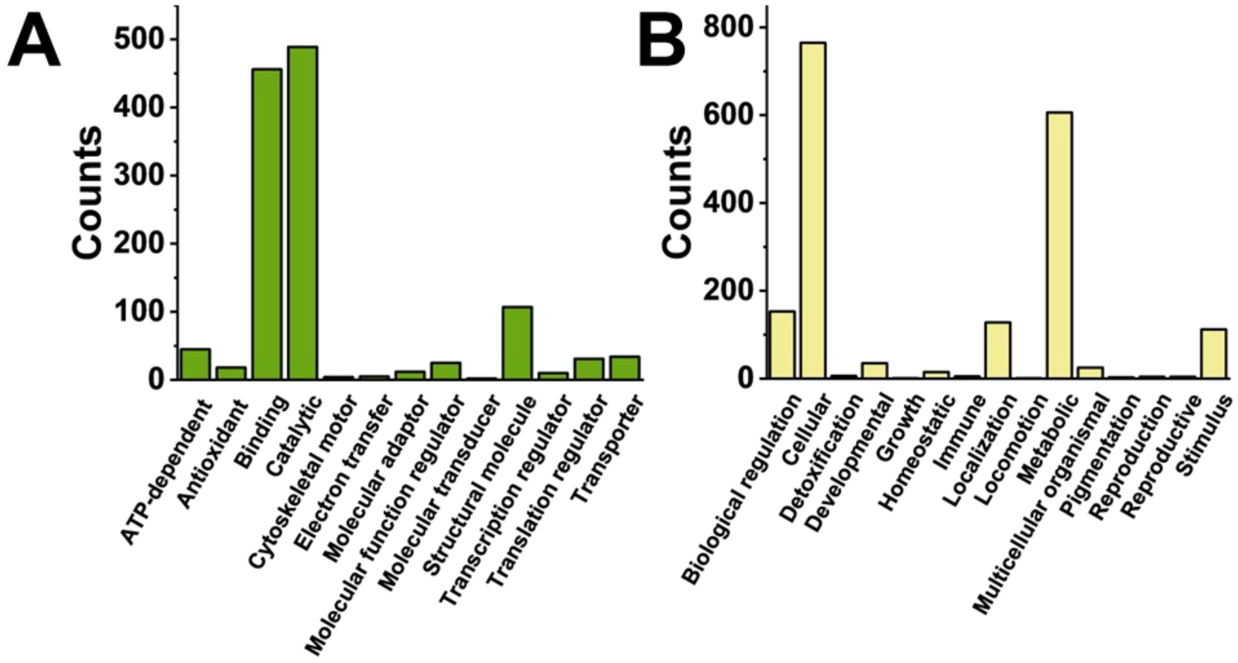

Figure S7. Interpretation of canonical knowledge for the proteins identified in the single *X. laevis* blastomeres. The proteins that were identified in ~2% of the single-cell proteome was matched to PantherDB 18.0[21]. Analysis of **(A)** molecular function and **(B)** biological processes among the detected proteins.

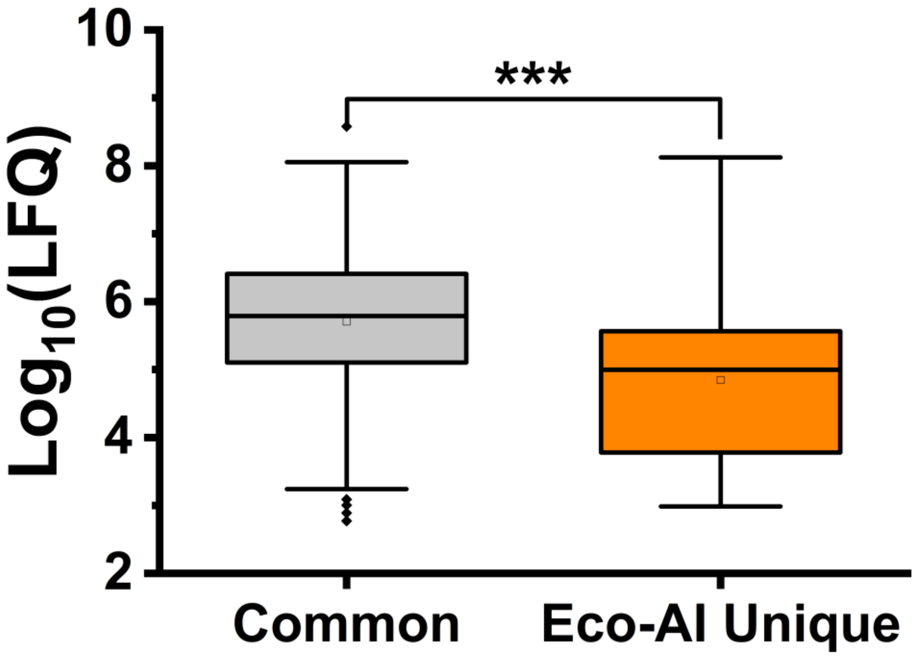

Figure S8. Comparison of Real-Time Eco–AI sensitivity against the DIA–Eco reference[10]. Real-Time Eco–AI identified proteins in the single *X. laevis* cells that occupied the lower domain of the measured concentration range via label-free quantification (LFQ). These differences were statistically significant, revealing notable sensitivity enhancement via Real-Time Eco–AI in this work. Key: *** $p < 0.001$ (Mann-Whitney *U* test).

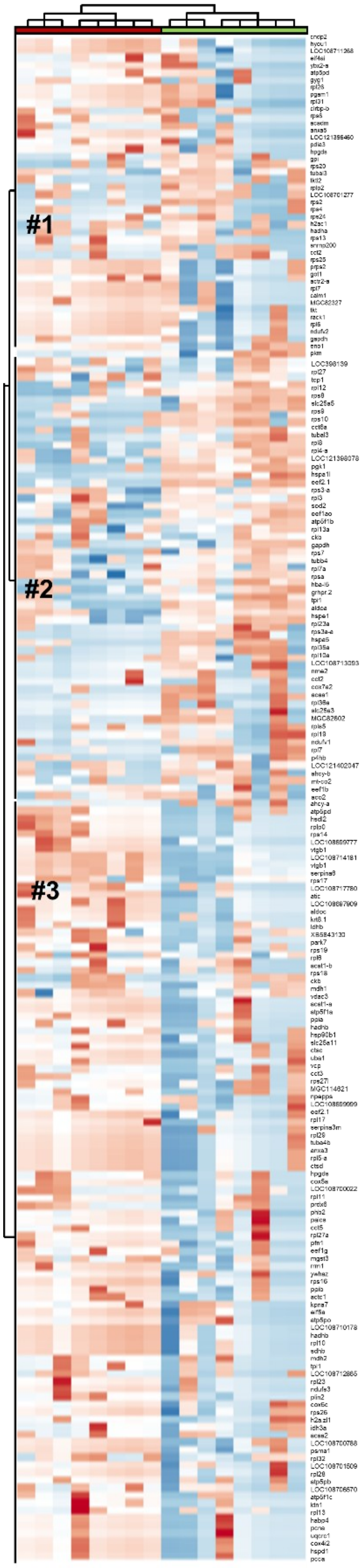

Figure S9. Close-up of the HCA-heat map of the top 200 most significantly differently differential proteins between the D11 and V11 clones at the stage-8 *X. laevis* blastula (**Fig. 5D**). The proteins are labeled with their corresponding gene names following the *Xenopus* naming nomenclature.

SI REFERENCES

[1] H. L. Sive, R. M. Grainger, R. M. Harland, *Early development of Xenopus laevis: a laboratory manual*, Cold Spring Harbor Laboratory Press, New York, **2000**.

[2] V. Kostiuk, M. K. Khokha, in *Amphibian Models of Development and Disease, Vol. 145* (Ed.: S. Y. Sokol), Elsevier Academic Press Inc, San Diego, **2021**, pp. 277-312.

[3] Z. B. Zhang, K. M. Dubiak, E. Shishkova, P. W. Huber, J. J. Coon, N. J. Dovichi, *Anal. Chem.* **2022**, *94*, 3254-3259.

[4] J. Newport, M. Kirschner, *Cell* **1982**, *30*, 675-686.

[5] S. L. Klein, *Dev. Biol.* **1987**, *120*, 299-304.

[6] P. D. Nieuwkoop, J. Faber, *Normal table of Xenopus laevis (daudin): a systematical & chronological survey of the development from the fertiilized egg till the end of metamorphosis*, Garland Science, **1994**.

[7] S. A. Moody, *Dev. Biol.* **1987**, *119*, 560-578.

[8] C. Lombard-Banek, S. A. Moody, M. C. Manzini, P. Nemes, *Anal. Chem.* **2019**, *91*, 4797-4805.

[9] B. W. Shen, L. R. Pade, S. B. Choi, P. Munoz-Llancao, M. C. Manzini, P. Nemes, *Front. Chem.* **2022**, *10*, No. 863979.

[10] B. Shen, L. Pade, P. Nemes, *J. Proteome Res.* **2025**, *23*, 692−703.

[11] L. L. Sun, G. J. Zhu, Z. B. Zhang, S. Mou, N. J. Dovichi, *J. Proteome Res.* **2015**, *14*, 2312-2321.

[12] C. Lombard-Banek, S. A. Moody, P. Nemes, *Angew. Chem. Int. Ed.* **2016**, *55*, 2454-2458.

[13] P. Nemes, I. Marginean, A. Vertes, *Anal. Chem.* **2007**, *79*, 3105-3116.

[14] V. Demichev, C. B. Messner, S. I. Vernardis, K. S. Lilley, M. Ralser, *Nat. Methods* **2020**, *17*, 41-44.

[15] Z. Q. Pang, Y. Lu, G. Y. Zhou, F. A. Hui, L. Xu, C. Viau, A. F. Spigelman, P. E. Macdonald, D. S. Wishart, S. Z. Li, J. G. Xia, *Nucleic Acids Res.* **2024**, *52*, W398-W406.

[16] R. Schmid, S. Heuckeroth, A. Korf, A. Smirnov, O. Myers, T. S. Dyrlund, R. Bushuiev, K. J. Murray, N. Hoffmann, M. S. Lu, A. Sarvepalli, Z. Zhang, M. Fleischauer, K. Durkop, M. Wesner, S. J. Hoogstra, E. Rudt, O. Mokshyna, C. Brungs, K. Ponomarov, L. Mutabdzija, T. Damiani, C. J. Pudney, M. Earll, P. O. Helmer, T. R. Fallon, T. Schulze, A. Rivas-Ubach, A. Bilbao, H. Richter, L. F. Nothias, M. X. Wang, M. Oresic, J. K. Weng, S. Bocker, A. Jeibmann, H. Hayen, U. Karst, P. C. Dorrestein, D. Petras, X. X. Du, T. Pluskal, *Nat. Biotechnol.* **2023**, *41*, 447-449.

[17] B. Shen, J. Chen, P. Nemes, *Anal. Chem.* **2024**, *96*, 15581–15587.

[18] I. Messana, D. V. Rossetti, L. Cassiano, F. Misiti, B. Giardina, M. Castagnola, *J. Chromatogr. B* **1997**, *699*, 149-171.

[19] O. V. Krokhin, G. Anderson, V. Spicer, L. L. Sun, N. J. Dovichi, *Anal. Chem.* **2017**, *89*, 2000-2008.

[20] T. Truong, K. G. I. Webber, S. M. Johnston, H. Boekweg, C. M. Lindgren, Y. R. Liang, A. Nydegger, X. F. Xie, T. M. Tsang, D. Jayatunge, J. L. Andersen, S. H. Payne, R. T. Kelly, *Angew. Chem. Int. Ed.* **2023**, *135*, No. e202303415.

[21] H. Y. Mi, D. Ebert, A. Muruganujan, C. Mills, L. P. Albou, T. Mushayamaha, P. D. Thomas, *Nucleic Acids Res.* **2021**, *49*, D394-D403.
